# An iterative strategy combining biophysical criteria and duration hidden Markov models for structural predictions of *Chlamydia trachomatis *σ^66 ^promoters

**DOI:** 10.1186/1471-2105-10-271

**Published:** 2009-08-28

**Authors:** Ronna R Mallios, David M Ojcius, David H Ardell

**Affiliations:** 1 School of Natural Sciences, University of California, PO Box 2039, Merced, CA 95344, USA; 2 University of California, San Francisco, 155 N Fresno Street, Fresno, CA 93701, USA

## Abstract

**Background:**

Promoter identification is a first step in the quest to explain gene regulation in bacteria. It has been demonstrated that the initiation of bacterial transcription depends upon the stability and topology of DNA in the promoter region as well as the binding affinity between the RNA polymerase σ-factor and promoter. However, promoter prediction algorithms to date have not explicitly used an ensemble of these factors as predictors. In addition, most promoter models have been trained on data from *Escherichia coli*. Although it has been shown that transcriptional mechanisms are similar among various bacteria, it is quite possible that the differences between *Escherichia coli *and *Chlamydia trachomatis *are large enough to recommend an organism-specific modeling effort.

**Results:**

Here we present an iterative stochastic model building procedure that combines such biophysical metrics as DNA stability, curvature, twist and stress-induced DNA duplex destabilization along with duration hidden Markov model parameters to model *Chlamydia trachomatis *σ^66 ^promoters from 29 experimentally verified sequences. Initially, iterative duration hidden Markov modeling of the training set sequences provides a scoring algorithm for *Chlamydia trachomatis *RNA polymerase σ^66^/DNA binding. Subsequently, an iterative application of Stepwise Binary Logistic Regression selects multiple promoter predictors and deletes/replaces training set sequences to determine an optimal training set. The resulting model predicts the final training set with a high degree of accuracy and provides insights into the structure of the promoter region. Model based genome-wide predictions are provided so that optimal promoter candidates can be experimentally evaluated, and refined models developed. Co-predictions with three other algorithms are also supplied to enhance reliability.

**Conclusion:**

This strategy and resulting model support the conjecture that DNA biophysical properties, along with RNA polymerase σ-factor/DNA binding collaboratively, contribute to a sequence's ability to promote transcription. This work provides a baseline model that can evolve as new *Chlamydia trachomatis *σ^66 ^promoters are identified with assistance from the provided genome-wide predictions. The proposed methodology is ideal for organisms with few identified promoters and relatively small genomes.

## Background

Identifying mechanisms that regulate gene expression in bacteria is essential for understanding and eventually controlling their pathogenicity. All known bacteria share a well conserved transcriptional holoenzyme, RNA polymerase (RNAP). The RNAP is comprised of a 3-subunit catalytic core plus a variable σ-factor subunit that provides DNA binding specificity. One of these σ-factors, σ^70 ^in *Escherichia coli*, participates in the transcription of a majority of genes including those with housekeeping functions.

*E. coli *is the best studied bacterial model with regard to promoter identification and prediction. As such, most promoter predictions are based upon the analysis of *E. coli *σ^70 ^promoter data. The earliest collections of *E. coli *σ^70 ^promoters revealed the -35 and -10 hexamer consensus motifs, TTGACA and TATAAT, that serve as recognition sites for the 2.4 and 4.2 domains of σ^70 ^[1-3].

Position weight matrices (PWMs) were the first models to quantify the hexamer motifs [4]. PWM models were expanded to quantify the variable-length spacer region between hexamers [5-7], which is important in orienting the hexameric motifs for interaction with the sigma binding factors [8]. Challenges encountered by PWM models include defining thresholds that are sensitive enough to include known promoters without predicting numerous false positives.

Most of the quantitative modeling efforts that ensued require training sets comprised of both positive and negative sequences. Artificial neural networks (ANNs) [9] have been trained on sequences of identified *E. coli *promoters and non-promoters. A hidden layer in the ANN architecture quantifies interactions among pairs and triplets of nucleotides. The resulting ANN scans and scores overlapping sequences, and reports a score in the range (0, 1) that indicates the likelihood of the sequence being a promoter. A time-delay neural network (TDNN) can combine two simple ANNs (one for each hexamer) with a variable-length spacer region [10].

Burden *et al *(2005) [11] measured the distance from the transcription start site (TSS) to the translation start site (TLS) of 771 *E. coli *promoters. They showed that the distribution peaks sharply around 30 nt, and that combining the TSS-TLS distribution with the NNPP2.2 TDNN [10] significantly enhances the specificity of the prediction.

In another machine learning approach that has been applied to model promoters, support vector machines (SVMs) were trained on *E. coli *promoter sequences of length 200 [12]. Although the SVM approach has the advantage of comprehensively quantifying the primary structure of the upstream region, it does not examine structures of higher order that motivate our approach.

A natural extension to PWMs that explicitly models an empirical spacing distribution between motifs is given by duration hidden Markov models (HMMs). Here "duration" refers to this explicit representation of a spacer length distribution, as opposed to the geometrically distributed lengths that are expected from components of profile hidden Markov models [13]. Although the variable-length spacer region between hexamers has been incorporated into promoter modeling and predictors before [5-7], none of these earlier efforts have integrated an explicit probabilistic representation of the spacer distribution into a reusable predictor as a duration HMM, which is arguably its most natural representation. On the other hand, while duration HMMs have been introduced into genome analysis (for example, in intron-exon modeling, see Winters-Hilt 2006 ), they have not to our knowledge been applied to modeling transcriptional or translational signals before.

Bacteria of the genus *Chlamydia *are obligate intracellular parasites that were genetically isolated from other bacteria nearly a billion years ago when they moved into their intracellular environment [14]. In humans, *Chlamydia *infections are responsible for infertility, blindness, arthritis and cardiovascular disease [15]. Because chlamydiae have an intracellular life-cycle, standard genetic techniques are often insufficient to study gene regulation [16]. Hence, only 30 to 40 promoters have been experimentally verified [16-19]. However, with a small genome of only about 1 Mbp and 895 genes, *Chlamydia trachomatis *(*CT*) makes a good candidate for *in silico *analysis.

Surveys of known bacterial promoters suggest that their structures are relatively diverse [8]. Additionally, established *CT *promoters display obvious differences from the established consensus hexamers of *E. coli *[16-19]. Although σ^66^, the *CT *analog of *E. coli *σ^70^, has DNA binding domains homologous to domains 2.4 and 4.2 in σ^70^, sequence based phylogenetic analysis of bacterial RNAP subunits has shown discernable evolutionary distance between the *CT *and *E. coli *RNAP subunits [20]. Therefore, it is plausible that an organism-specific model is appropriate for *CT*.

Phylogenetic footprinting takes advantage of relative conservation of motifs among related species. Grech *et al *(2007) [17] developed an algorithm that combines *E. coli *trained PWMs and chlamydial phylogenetic footprinting. *CT *upstream regions are screened with the PWMs and the potential promoter hexamers are filtered with an algorithm that accepts only conserved sequences in a consensus of *C. trachomatis*, *C. pneumoniae *and *C. caviae*. Although this is a promising approach, because they used an *E. coli *trained PWM, their results may be strongly influenced by prior expectations that all bacterial promoters are structured as in *E. coli*. We believe that more development is needed in *ab initio *approaches for predicting promoters using sequence information directly from the organism under study (and perhaps from close phylogenetic relatives) in combination with biophysical metrics that derive from known models about the biology of transcription in general.

This study aims to develop *CT *promoter models using only known *CT *promoters. To do so, it considers DNA stability and topological features of the upstream region as well as RNAP σ-factor/DNA binding. As Hertz and Stormo (1996) [5] aptly wrote "... the polymerase needs to bind the DNA, open the DNA, initiate transcription, and release the promoter for elongation." The TDNNs and SVMs that consider extended promoter sequences are addressing this issue from a sequence perspective. This study utilizes measures that have been developed to quantify stability and other aspects of DNA structure. Evidence from the profiling of DNA curvature, bendability, twist, stability and propensity for stress-induced destabilization in *E. coli*, *B. subtilis*, *C. trachomatis*, plants and vertebrates [21-23] suggests that there are peaks for these measures near the TSS. Here we use a stochastic model building procedure that allows for the combination of relevant predictive measures selected from RNAP σ-factor/DNA binding propensity, as quantified by duration HMMs, and structural features of the upstream region, as quantified by biophysical metrics.

## Methods

### Stochastic Model Building

Stepwise Binary Logistic Regression (SBLR) [24,25], as implemented in SPSS version 17.0 statistical software (SPSS Inc., Chicago, IL), selects an optimal set of independent variables (continuous and/or categorical) to classify observations into two populations. Logistic regression does not assume a linear relationship between the dependent and independent variables, normal distributions, or homoscedasticity (equal variances). It does, however, assume independence of observations. We address this requirement in a separate section describing the selection of non-redundant observations.

The mathematical model (prediction equation) fitted by SBLR has the form



where i is the number of steps, v_1 _through v_i _are the predictor variables selected, and b_0 _through b_i _are coefficients determined by the analysis.

**u **is the logit for the dependent variable, which means that



Here, the event is class membership. When P denotes the prob(class membership), the equation can be rewritten as



Selecting a cutoff for P, most commonly 0.5, converts P into a classifier. When 0.5 is the probability threshold, e^**u **^= 1 and the classification threshold for **u **is 0. The effectiveness of a model can be evaluated by its ability to correctly classify the training data.

The SPSS SBLR analysis procedure provides many user-defined options. We selected the Forward Conditional stepwise procedure for all analyses. At each step, a score statistic is calculated for each variable excluded from the model. The score statistic is based on Maximum-Likelihood Estimation criteria and is asymptotically distributed as a χ^2 ^variable [25]. The variable with the highest significant χ^2 ^value is entered into the model. If no significant variables remain, then the procedure stops with the current model. Similarly there is a mechanism for stepwise removal. After a new model has been generated, score statistics are calculated for all variables in the model. If the p-value for any variable in the model is greater than the probability for stepwise removal, then the variable is removed from the model. We retained the default probabilities for stepwise entry (.05) and removal (.10), thus ensuring that the significance of all model variables is less than 0.10.

### Potential Observations and Dependent Variable

A significant challenge for bioinformaticians is to model data that has been collected by multiple laboratories using different assays, protocols and equipment. This phenomenon is compounded in the study of *CT *where the organism is metabolically active only inside an infected host-cell. One way to minimize the use of conflicting and/or controversial data is to rely upon reviews written by informed biologists. For this reason, we consulted the reviews of Mathews & Timms (2006) [19] and Tan (2006) [16] to compile a list of 16 experimentally verified σ^66 ^promoters. To this list we added 13 promoters that were experimentally verified by Grech *et al *(2007) [17] and Hefty *et al *(2007) [18] after the previously cited reviews were written. For the purposes of this study, we consider these 29 sequences to be the known *CT *σ^66 ^promoters.

Table 1 describes the 29 experimentally verified σ^66 ^promoters from 27 genes that form the basis of the training set for this study. We derived potential observations for analysis according to the following procedure:

**Table 1:** 29 experimentally verified σ^66 ^promoters.

CT	Name	To TLS^a^	Ref^b^	-35 Hex	Spacer (16-20)	-10 Hex	h PI^c^
CT046	*hctB*	107	M	TGGTTA	GTTTTTAATAAAAAGT(16)	TAAAAA	16

CT062	*tyrS*	62	G	TTGCTA	TAAAAAGAACAGGATAGA(18)	TAAGAT	8

CT080	*ltuB*	68	M,T	TTATGA	AAAACAATTTTTTAATT(17)	TAAAAT	24

CT091	*yscU*	68	H	TTGAGA	AAAACATTTATATACGG(17)	TAACTT	8

CT098	*rs1*	69	M,T	TTGCCT	TTTTTAAGGTGAATATT(17)	TACACT	3

CT111	*groES*	129	M,T	TTGCAA	AAAAGCGAGGACTTTGC(17)	TATCGT	1

CT286	*clpC*	64	G	TTGCAT	CATTATCATAAATGTCG(17)	TATATG	8

CT322	*tuf*	296	M,T	TTGATA	ATAATCCGCGTCTGAAGT(18)	TACTAT	3

CT323	*infA*	145	M,T	TTGACA	TTTTCTGTTTAGTCGA(16)	TATAAT	3

CT377	*ltuA*	74	M,T	TGCAGA	GTTTTTATTTTAAATATGT(19)	TATAAT	16

CT394	*hrcA*	40	M,T	TTGACC	AGTGGAGACGGTTTTCT(17)	TATAAT	16

CT439m	*rpsL*	67	G	TTGCAA	ACAAAGATATTCTTATTC(18)	TATATT	3

CT442	*crpA*	66	M	GGGTTT	TTGAAAAAAACAAGTGTTT(19)	GTGTAG	16

CT444a	*omcA*	127	M,T	TTGATA	TAATTTTTATTTTATAA(17)	TGTAAT	16

CT444b	*omcA*	61	M,T	AATTGC	TTTTATCGATAAAAGAAAC(19)	TTCAAG	16

CT518	*rl14*	198	M	CTGTTG	TTGTTCGAGTCGAAAGGG(18)	TATACT	3

CT557	*lpdA*	162	H	TTGAGA	TTTTATCCACCCAGATG(17)	TACAAC	8

CT559	*yscJ*	52	G	TTGGCA	CTAATCTCCCCATTTGC(17)	TATGGT	16

CT576	*lcrH_1*	75	H	TTGTTA	AATCAGATCGTTAGAATT(18)	TAATAT	16

CT596	*exbB*	63	G	TTGGTT	CTATACAAGAAATTTGT(17)	TAGGAT	3

CT665	-	98	H	TTGTAT	CTTTTTAGAACGGGAAGGG(19)	TTGAAA	8

CT674	*yscC*	119	H	TTGCAA	GATAGAGGGCAAATAGA(17)	TATATT	16

CT681a	*ompA*	282	M,T	TATACA	AAAATGGCTCTCTGCTT(17)	TATTGC	8

CT681b	*ompA*	60	M,T	GTGCCG	CCAGAAAAAGATAGCGAG(18)	CACAAA	8

CT701	*secA_2*	57	M	TGTATA	GGCGCCTTTAAATAAGAGGG(20)	TAGGTT	8

CT708	-	66	G	TTGATT	TAGCGGAAGTAAAAAGG(17)	TACAAG	16

CT743	*hctA*	83	M,T	TTGCAT	GAATTTGAACAAACAAAC(18)	TAATTA	24

CT752	*efp_2*	62	G	TGGACA	AAGCTTAGAAGAGAACGA(18)	TAACAT	8

CT863	-	71	H	TTGCAT	GAAAAATACTTTTTAGA(17)	TAAGTT	16

^a^nt distance from the lead nt of the -35 hexamer to the TLS.

^b^References: M: Mathews & Timms [19]; G: Grech *et al *[17]; H: Hefty *et al *[18]; T: Tan [16]

^c^hour Post Infection of transcriptional activation [31]

1. Files containing the *CT *genome (NC_000117.fna) and genome table (NC_000117.ptt) were retrieved from the NCBI website, , on July 2, 2007 and last modified by NCBI on January 23, 2007. An R script was written to extract the 600 nt upstream regions of all 895 protein-coding genes as annotated in the genome table. The regions were verified at several stages throughout the research with other sources, including a *CT *genome database previously available from Los Alamos National Laboratories and comparable prediction algorithms. Table 1 displays the distance from promoter to TLS for all training set genes. Since the maximum distance is 296 nt, 325 nt was set as the upper limit for data analysis. Then, the upstream region was defined as 600 nt to allow for biophysical structures 275 nt upstream from a predicted promoter.

2. For each of the 27 genes listed in Table 1, the 600 nt upstream region was parsed into overlapping sliding window sequences of length 32 (6 nt for each hexamer and maximum spacer of 20 nt) and step-size 1. Each subsequence (SEQ32) was labeled according to its parent gene and position occupied in the upstream region: e.g. the first SEQ32 was labeled CT046_600 because the initial nt is found 600 nts upstream from the CT046 TLS.

3. The dependent variable, PROMOTER, was assigned a 1 if a promoter sequence listed in Table 1 was totally contained in SEQ32, and 0 otherwise. Thus, 1's identify potential promoter observations and 0's identify potential non-promoter observations.

4. Cases with upstream positions ≥ 40 and ≤ 325 were selected as potential observations to restrict the analysis to the range of the training set data. The upper bound is 30 nt upstream of the furthest upstream training set promoter and the lower bound is equal to the furthest downstream training set promoter.

### Independent Variables

The primary variable for promoter prediction is the pattern that characterizes the binding between the RNAP σ-factor and DNA. Here we use duration HMMs to describe and quantify RNAP-σ^66^/DNA binding. After a set of known promoters is used to train a duration HMM, the duration HMM scans a new sequence to identify the hexamer-spacer-hexamer subsequence that scores the highest with regard to potential binding. The variable HMM_SCORE is assigned the score associated with the highest scoring subsequence, while the variable START denotes the position of the lead nucleotide in the -35 hexamer and END denotes the position of the last nucleotide of the -10 hexamer.

Specifically, a training set of promoter sequences was placed in the file ts.txt. The initial ts.txt contained the contents of Table 1, columns "-35 Hex", Spacer and "-10 Hex". This file was supplied as input to **durahmmer **(Ardell D.H., in preparation) which was used to create a duration HMM with the command: **durahmmer **-5 6 -3 6 -s 16 -S 20 -p 1 -u 28.5:21.5:21.5:28.5 -C ts.txt > ts.hmm. The options to the command specify the following model parameters: 6 matched states (hexamers) at the 5' and 3' sequence ends; minimum and maximum spacer lengths of 16 and 20 respectively; a background compositional model of 28.5% A, 21.5% C, 21.5% G, and 28.5% T; and spacers should be modeled to have their empirical composition in the training set (which in this case was: 38% A, 12% C, 17% G, 33% T). The program **durahmmer **produces a valid HMMer 2.3.2 [13] model file representing a duration HMM. For the final model of this study, the model file and the input data file are provided as Additional Files 1 and 2. All 16,200 SEQ32 observations from the 27 genes were placed in the file all.txt so that optimal promoters and HMM scores could be calculated by **hmmsearch **[13] with the command: **hmmsearch **-E 9000 ts.hmm all.txt. We ran **hmmsearch **with a high E-value because we were interested in combining the score of the maximum scoring hit with other metrics in a composite procedure regardless of its magnitude.

In combination with the duration HMM model score described above, we also used the following biophysical metrics of promoter position and structure as possible independent variables for the SBLR model:

1. POSITION, which indicates the location of SEQ32 in the upstream region relative to the TLS. For CT046_101, POSITION = 101.

2. Measures of curvature (CURVE) [26] and %GC content (GC) for each 600 nt upstream region, which were determined by the online bend.it Server  with a window-size of 32.

3. Free energy change (ΔG) of DNA melting (parameter #33 [27], dinucleotide, window size 2), bendability (parameter #31 [28], trinucleotide, window size 3) and twist angle (parameter #44 [29], dinucleotide, window size 2), which were determined for each 600 nt upstream region by the online plot.it Server . All measurements were then averaged over each SEQ32. ΔG always has a negative sign and is interpreted as greater values having lower stability. For statistical analysis this variable was transformed by STABLE = -ΔG so that the sign is always positive and the interpretation is that larger values have greater stability. Stability is also of interest in the immediate downstream region, so positions 27-37 (STABLE27_37) and 1-37 (STABLE1_37) were quantified. Since the bendability measure increases with rigidity, it was renamed RIGID. The twist angle measurement, TWIST, was not transformed.

4. Possible times of expression onset include 1, 3, 8, 24 and 40 hours post infection (h PI). Mutually exclusive binary variables H1, H3, H8, H16, H24 and H40 were created to mark time of expression onset.

5. Stress-induced DNA duplex destabilization (SIDD) quantification utilizes structural and energetic properties of DNA to measure the propensity for strand separation under negative superhelical stress [22]. A low SIDD score indicates a high propensity for strand separation. SIDD measurements were determined by the WebSIDD server [30] with default parameters except for Open Region Size = 63. Because Niehaus *et al *[31] have shown a time dependent response to chlamydial DNA supercoiling, interactions between the time of expression onset and SIDD were included [32]. The SIDD/hour of onset interaction is quantified by SIDD_H# = SIDD*H#.

6. For variables based on the entire SEQ32, lagged variables were created for the four non-overlapping upstream subsequences of length 32: e.g. for CT046_100, CURVE_L32 was set equal to the CURVE value of CT046_132, CURVE_L64 was set equal to the CURVE value of CT046_164; CURVE_L96 was set equal to the CURVE value of CT046_196; and CURVE_L128 was set equal to the CURVE value of CT046_228.

### Selection of Non-redundant Observations from Potential Observations

As mentioned earlier, SBLR assumes independent observations. To address this requirement, we select for analysis a subset of the overlapping potential observations that are non-redundant with respect to the pair of hexamers that are most likely to bind the RNAP σ-factor.

Table 2 displays the first six columns of a portion of the data file used for analysis. Each potential observation occupies a row. A row includes: the SEQ32 label (SEQ_ID); the SEQ32 literal sequence (SEQ32); the score of the optimal HMM instance in SEQ32 (HMM_SCORE); the position of the lead nt in the -35 hexamer of the optimal HMM instance (START); the position of the last nt in the -10 hexamer of the optimal HMM instance (END); and PROMOTER as previously defined.

**Table 2:** Selecting rows with END = 32 (*) ensures non-redundant observations with regard to hexamers and HMM_SCORE.

SEQ32_ID	PRO-MOTER	START	END	HMM_ SCORE	SEQ32:bold italics locates optimal HMM instance
CT046_117	0	4	* 32	-5.9	TAA***TTGTGT***GTGGTTAGTTTTTAATA***AAAAGT***

CT046_116	0	3	31	-5.9	AA***TTGTGT***GTGGTTAGTTTTTAATA***AAAAGT***T

CT046_115	0	2	30	-5.9	A***TTGTGT***GTGGTTAGTTTTTAATA***AAAAGT***TA

CT046_114	0	1	29	-5.9	***TTGTGT***GTGGTTAGTTTTTAATA***AAAAGT***TAA

CT046_113	0	2	29	-13.7	T***GTGTGT***GGTTAGTTTTTAATAA***AAAGTT***AAA

CT046_112	0	1	* 32	-11.4	***GTGTGT***GGTTAGTTTTTAATAAAAAG***TTAAAA***

CT046_111	1	5	* 32	-2.1	TGTG***TGGTTA***GTTTTTAATAAAAAGT***TAAAAA***

CT046_110	1	4	31	-2.1	GTG***TGGTTA***GTTTTTAATAAAAAGT***TAAAAA***C

CT046_109	1	3	30	-2.1	TG***TGGTTA***GTTTTTAATAAAAAGT***TAAAAA***CT

CT046_108	1	2	29	-2.1	G***TGGTTA***GTTTTTAATAAAAAGT***TAAAAA***CTA

CT046_107	1	1	28	-2.1	***TGGTTA***GTTTTTAATAAAAAGT***TAAAAA***CTAA

CT046_106	0	3	31	-11.9	GG***TTAGTT***TTTAATAAAAAGT***TAAAAA***CTAAC

CT046_105	0	2	30	-11.9	G***TTAGTT***TTTAATAAAAAGT***TAAAAA***CTAACC

CT046_104	0	1	* 32	-7.6	***TTAGTT***TTTAATAAAAAGTTAAAAAC***TAACCA***

CT046_103	0	4	* 32	-7.8	TAG***TTTTTA***ATAAAAAGTTAAAAACT***AACCAT***

CT046_102	0	3	31	-7.8	AG***TTTTTA***ATAAAAAGTTAAAAACT***AACCAT***T

CT046_101	0	2	30	-7.8	G***TTTTTA***ATAAAAAGTTAAAAACT***AACCAT***TT

If we select only those cases where END = 32, we eliminate all of the redundant optimal HMM hexamer pairs while retaining most optimal HMM instances (information). Table 2 demonstrates how this selection ensures that neighboring optimal HMM instances that match are included only once. Six potential observations, CT046_111 through CT046_106, all contain the verified promoter with hexamer pair TGGTTA and TAAAAA. Consequently, they all have PROMOTER = 1 and HMM_SCORE = -2.1. But only CT046_111 has END = 32 and is selected to represent the verified CT046 promoter. Similarly, only CT046_117 represents the maximal non-promoter hexamer pair TTGTGT and AAAAGT with score = -5.9. This process incidentally aligns each selected SEQ32 such that the optimal downstream hexamer is at the far right end.

This selection process does not eliminate overlapping sequences, but it does eliminate overlapping likely binding sites. CT046_111 and CT046_112 overlap a great deal. However, the last hexamer of CT_046_111 (TAAAAA) is not present in CT046_112 and the first hexamer of CT_046_112 (GTGTGT) does not appear in CT046_111.

It should be noted that although each training set gene begins with the same number of potential observations, this selection process causes the number of selected non-redundant observations to differ among genes. Each gene starts with around 5 potential observations with PROMOTER = 1 for each verified promoter, and around 325-40-5 = 280 potential observations with PROMOTER = 0. However, selection for non-redundant observations always results in the number of designated non-promoters being reduced to approximately 90.

While selecting sequences with non-redundant HMM_SCORES does mitigate the problem of dependent observations, it may not entirely eliminate it. While there are numerous studies that affirm the robustness of Baysian Discriminant Analysis with regard to violating the assumptions of a linear relationship between the dependent and independent variables, normal distributions, and homoscedasticity [33], we could not find similar studies regarding the robustness of logistic regression. An alternative to the current analysis would be to use Stepwise Discriminant Analysis, knowing that we are violating some assumptions.

There are versions of logistic regression, including generalized estimating equations (GEE) [25], that are specifically designed for correlated data such as longitudinal studies. In these procedures there are subject variables and within-subject variables. It might be possible to force this study data into such a format, but as yet there are no readily available stepwise procedures to scan multiple possible predictors. A final alternative would be to select non-overlapping sequences with the penalty of losing information and perhaps introducing a selection bias.

SBLR is a procedure for model identification. It is only after a model has been identified that it can be evaluated for independence. Given that, we elected to analyze the non-redundant observations with SBLR and then examine the error terms for independence. In Time Series Analysis (which this analysis most resembles), this is done by checking that the error term is normally distributed with zero mean, and that autocorrelations and partial autocorrelations of the error term are not significant [34].

### Iterative Modeling Strategy

**Figure 1:**
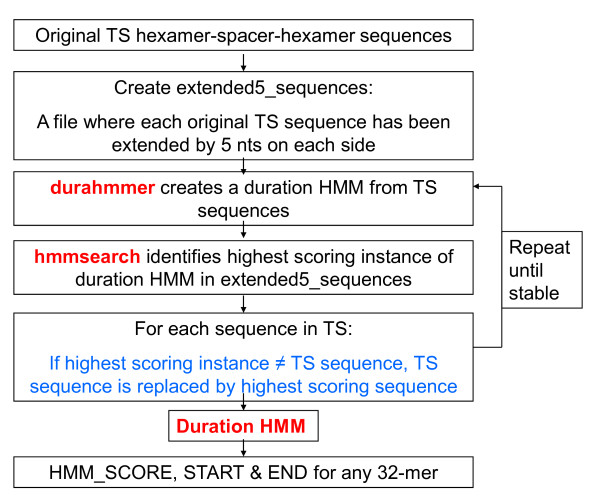
**Flowchart of duration HMM iteration**.

**Figure 2:**
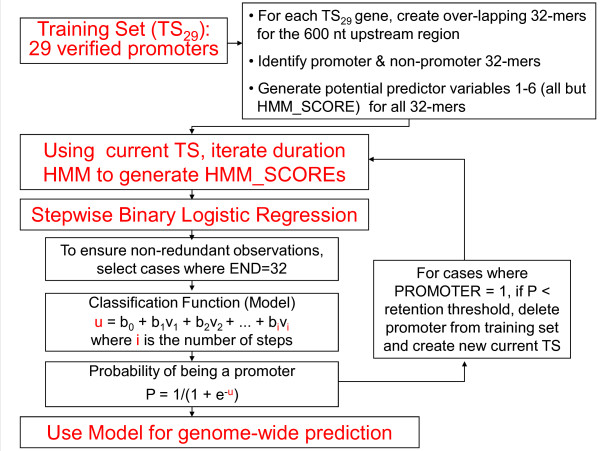
**Flowchart of Stepwise Binary Logistic Regression iteration**.

Sources of error that could lead to misclassification include (i) imprecise laboratory procedures in defining and identifying promoters (including false positive promoters), (ii) presence of more than one promoter population, (iii) failure to include relevant predictor variables, and (iv) random variation. To minimize the first two error sources, an iterative strategy was developed. Duration HMM iteration (Figure 1) addresses error source (i), while SBLR iteration (Figure 2) addresses source (ii).

#### Duration HMM Iteration (Figure 1)

Minor modifications in the configuration of the training set promoters can improve classification accuracy. To accomplish this, we allowed each promoter to vary within a neighborhood that extends the sequence by 5 nts on each side. A limit of 5 nts ensures that a modified hexamer will not locate completely outside of the original promoter sequence. For example, when the promoter CT377 is extended, it becomes **TTGTT**TGCAGAGTTTTTATTTTAAATATGTTATAAT**CTGTC**, with the bolded nts marking the extensions. Initially, a duration HMM is determined by the original, non-extended, promoter training set. Then the set of extended promoters is searched for the highest scoring instance of the duration HMM in each extended sequence. If a high-scorer is not the same as the original promoter, it replaces the original in the training set. The iteration continues until stabilization. For the final model, CT377 was modified to TTGCAGAGTTTTTATTTTAAATATGTTATAAT.

#### SBLR Iteration (Figure 2)

Deletion and subsequent replacement of members of the training set can eliminate promoters that are likely to be members of a different promoter population. This is accomplished via the iterative scheme diagrammed in Figure 2. Initially, the complete set of 29 verified promoters determines the duration HMM and the independent observations selected for SBLR analysis. SBLR delivers a mathematical model that produces a predicted probability of class membership (P) for each observation. A threshold on P of .5 is used to classify each observation as a predicted promoter or non-promoter.

For those 29 cases where PROMOTER = 1, we also use the value of P to determine when a promoter appears to be an outlier and should be eliminated from the training set. After observing the 29 probabilities, a retention threshold on P between 0 and .1 is established. If a training gene has only one identified promoter and that promoter has a P less than the retention threshold, then all observations for that gene are deleted from the analysis. Similarly, if a training set gene has two identified promoters and they are both selected for deletion, all observations for that gene are deleted. However, if a training set gene has two identified promoters and only one is selected for deletion, all upstream observations for that gene remain in the analysis dataset and only observations within the remaining promoter are assigned PROMOTER = 1.

Modifying the training set in any way necessitates the determination of a new duration HMM, which in turn determines which observations will be aligned such that END = 32 and subsequently included in the next SBLR analysis. The iteration process continues until the training set stabilizes.

### Stratified K-fold Cross-Validation

Once the final training set and model are selected, it is necessary to validate the model to ensure against over-fitting and to allow for comparisons with algorithms trained on other datasets. In the case of dichotomous classification, stratified K-fold cross-validation [35] partitions the training set into K subsamples such that each subsample has approximately the same proportions of class membership. Here we designate each training gene as a subsample; hence K equals the number of genes in the training set. Then, one gene (1-2 promoters and approximately 90 non-promoters) is retained as a validation set while the remaining genes are used as training data. Evaluation measures are calculated by aggregating the results of each validation set.

### Comparable Algorithms

The following three algorithms were used to compare performance and to identify co-predictions with the model developed in this study: NNPP2.2, TSS-PREDICT, and Footy. NNPP2.2 [10] is an online time-delay neural network that is accessible for promoter predictions at . We used the following options: organism = prokaryote and minimum promoter score = 0.95 to define promoters in the 325 nt upstream region of all *CT *genes. For the support vector machine algorithm TSS-PREDICT [12], the top two ranking predictions for each *CT *gene are posted as supplementary material at doi:10.1016/j.combiolchem.2008.07.009. The 42 *CT *promoters predicted by Footy [17], an algorithm that utilizes phylogenetic footprinting, are reported directly in the publication that describes the algorithm.

R scripts scanned the promoters predicted by NNPP2.2 and TSS-PREDICT for matches with the promoters predicted by the study model. An NNPP2.2 match was declared when the study prediction was contained within the 50 nt NNPP2.2 prediction. A TSS_PREDICT match was declared when the TSS_PREDICT predicted hexamer pair was contained within the study prediction.

## Results

### Finding the Best Model

The initial model, M0, utilizes the initial training set of 29 promoters with observations from their 27 parent genes. The duration HMM model converged after one iteration, modifying the alignment of 7 promoters. For all models, Table 3 reports the variables that were selected for the model and evaluation measures. If TP = true positive, FP = false positive, TN = true negative and FN = false negative, then sensitivity or recall = TP/(TP+FN), specificity = TN/(FP+TN), positive predictive value (PPV) or precision = TP/(TP+FP), negative predictive value (NPV) = TN/(FN+TN), and accuracy = (TP+TN)/(TP+TN+FP+FN). The total number of observations for each model differs according to the promoter training set being used.

**Table 3:** Models produced by Stepwise Binary Logistic Regression Iteration and M2 Cross-Validation.

SBLR Model	M0	M1	M2	M3	M2 Cross-Validation
Training Set Deletion	none	CT665 CT681a CT681b CT743	CT665 CT681a CT681b	CT681a CT681b	CT665 CT681a CT681b

Variables in Model^a^	+HMM_SCORE +STABLE1_37 -POSITION +CURVE_L32 -GC_L128 +RIGID_L96 +CURVE	+HMM_SCORE +STABLE1_37 -GC_L32 -POSITION +CURVE_L32 -CURVE_L64 -GC_L128 +TWIST	+HMM_SCORE +STABLE1_37 -POSITION +CURVE_L32 -STABLE_L32 +SIDD_H24 -CURVE_L128 -SIDD_L128 +RIGID_L96	+HMM_SCORE +STABLE1_37 -POSITION +CURVE_L32 -STABLE_L32 -STABLE27_37 +CURVE	

Sensitivity or Recall	19/29 (0.655)	25/25 (1.0)	26/26 (1.0)	25/27 (0.926)	23/26 (0.885)

Specificity	2426/2428 (0.999)	2083/2083 (1.0)	2226/2226 (1.0)	2322/2323 (1.0)	2215/2226 (0.995)

PPV or Precision	19/21 (0.905)	25/25 (1.0)	26/26 (1.0)	25/26 (0.962)	23/34 (0.676)

NPV	2426/2436 (.996)	2083/2083 (1.0)	2226/2226 (1.0)	2322/2324 (0.999)	2215/2218 (0.999)

Accuracy	2445/2457 (0.995)	2108/2108 (1.0)	2252/2252 (1.0)	2347/2350 (0.999)	2238/2252 (0.994)

AUC^b^	0.995	1.0	1.0	0.999	0.992

^a^The variables are listed in order of entrance into the model and the sign indicates the sign of the coefficient.

^b^ROC analysis Area Under the Curve

For model M0, 19 of the 29 promoters were classified correctly, with 2 false positives. There is always the possibility that these are yet to be recognized promoters, but at this point they are counted as misclassifications. For the 10 verified promoters that were missed, the predicted probabilities ranged from 0.001 to 0.42. Since a natural separation appeared to between 0.07 and 0.10, P = 0.08 was selected as the retention threshold and promoters CT665, CT681a, CT681b and CT743 (along with all observations from their parent genes) were deleted from the training set for the next model, M1.

The duration HMM model for M1 converged after two iterations, modifying the alignment of 5 promoters. Table 3 shows that M1 classified the modified training set perfectly, indicating that perhaps too many promoters had been deleted from the original training set. The retention threshold was reset to 0.07 and CT743 was reinstated for model M2.

The duration HMM model for M2 converged after one iteration. Table 4 displays the alignments of the 6 promoters that were modified. M2 also classified the modified training set perfectly. Again the results indicated that the next model, M3, should reset the retention threshold to 0.06 and reinstate CT665. However, Table 3 reports that M3 is not as good as models M1 and M2 because of classification errors.

**Table 4:** M2 duration HMM sequence alignment modifications.

CT	Name	To TLS	-35 Hex	Spacer (16-20)	-10 Hex
CT323	*infA*	145	TTGACA	TTTTCTGTTTAGTCGA(16)	TATAAT

		149	TTGTTT	GACATTTTCTGTTTAGTCGA(20)	TATAAT

CT377	*ltuA*	74	TGCAGA	GTTTTTATTTTAAATATGT(19)	TATAAT

		75	TTGCAG	AGTTTTTATTTTAAATATGT(20)	TATAAT

CT442	*crpA*	66	GGGTTT	TTGAAAAAAACAAGTGTTT(19)	GTGTAG

		60	TTGAAA	AAAACAAGTGTTTGTG(16)	TAGACT

CT444b	*omcA*	61	AATTGC	TTTTATCGATAAAAGAAAC(19)	TTCAAG

		59	TTGCTT	TTATCGATAAAAGAAAC(17)	TTCAAG

CT518	*rl14*	198	CTGTTG	TTGTTCGAGTCGAAAGGG(18)	TATACT

		195	TTGTTG	TTCGAGTCGAAAGGGTA(17)	TACTCG

CT701	*secA_2*	57	TGTATA	GGCGCCTTTAAATAAGAGGG(20)	TAGGTT

		61	TTGTTG	TATAGGCGCCTTTAAA(16)	TAAGAG

Given two models, one training set a subset of the other, that both classify their respective training sets with 100% accuracy, we reasoned that the model trained on the largest set would provide the most sensitive genome-wide prediction. Thus, M2 was selected as the best and final model because of the perfect classification with the largest training set. The complete data file used to build M2, Additional File 3, is supplied so that others may replicate or modify the model.

Finally, the error terms of M2 were checked for independence. Residuals, PROMOTER - P, were calculated for all selected observations and shown to be normally distributed with zero mean. Additionally, the autocorrelations and partial autocorrelations of the residuals were not significant. Thus, the independence assumption of SBLR was not violated by this model.

Aggregated results of the stratified K-fold (25-fold) M2 cross-validation are reported in the last column of Table 3. For the 25 genes in the M2 training set, 3 promoters (CT322_298, CT743_085, and CT752_064) were not identified (sensitivity = 0.885) and there were 11 false-positive predictions (precision = 0.676). The incorrect classifications are most likely due to incomplete representation of the sample space, but may indicate additional populations or absent predictors.

Table 5 compares the performance of the stratified K-fold cross-validation performance of the M2 model with that of comparable algorithms when predicting promoters in the 25 cross-validation genes. The tally is in the form hits/predictions/gene. For NNPP2.2, a prediction was considered a hit if the hexamer pair in Table 1 was fully contained in the 50-mer NNPP2.2 prediction using a threshold of 0.95. The last two rows of the table show the cumulative sensitivity and precision of each prediction algorithm. M2 cross-validation is the most sensitive (0.885), while Footy is the most precise (1.0). Table 6 reports the hits and misses for the 2 genes that were not used in the M2 model. The only hit was scored by NNPP2.2, with 2 accompanying false positives.

**Table 5:** Comparison of M2 Cross-Validation and predictions of comparable algorithms for 25 training set genes.

CT	HMM2 SCORE	M2 Cross-Validation	NNPP2.2	TSS-PREDICT	Footy
CT046	-1.6	1/1	0/4	0/2	0/0

CT062	4.0	1/2	0/0	1/1	1/1

CT080	0.5	1/2	1/4	0/2	0/0

CT091	1.3	1/3	1/1	1/1	0/0

CT098	3.7	1/1	0/1	1/2	1/1

CT111	-1.0	1/1	1/3	0/2	1/1

CT286	1.2	1/1	1/2	1/1	1/1

CT322	-2.1	0/0	0/0	0/2	0/0

CT323	1.6	1/1	1/3	1/1	1/1

CT377	5.3	1/2	1/3	1/1	0/0

CT394	-1.5	1/1	1/2	1/1	0/0

CT439m	1.8	1/1	0/3	0/0	1/1

CT442	-0.9	1/2	1/1	1/1	0/0

CT444	3.2	2/5	2/5	1/2	0/0

CT518	-4.2	1/1	0/0	1/1	0/0

CT557	-3.4	1/1	0/1	1/1	0/0

CT559	-2.7	1/1	1/1	0/2	1/1

CT576	0.6	1/2	1/3	1/2	0/0

CT596	0.5	1/1	0/1	0/2	1/1

CT674	4.0	1/2	1/2	0/0	0/0

CT701	-3.3	1/1	1/2	0/2	0/0

CT708	2.6	1/1	1/2	1/1	1/1

CT743	-3.8	0/0	0/2	1/5	0/0

CT752	-3.5	0/0	1/1	0/2	1/1

CT863	4.0	1/1	1/1	1/1	0/0

					

Sensitivity		23/26 (0.89)	17/26 (0.65)	15/26 (0.58)	10/26 (0.39)

Precision		23/34 (0.68)	17/48 (0.35)	15/38 (0.40)	10/10 (1.0)

**Table 6:** Comparing predictions of M2 and other algorithms for 2 training set genes not in M2 training set.

CT	M2	NNPP2.2	TSS-PREDICT	Footy
CT665	0/1	1/3	0/2	0/0

CT681	0/1	0/1	0/2	0/1

### Model Interpretation

**Figure 3:**
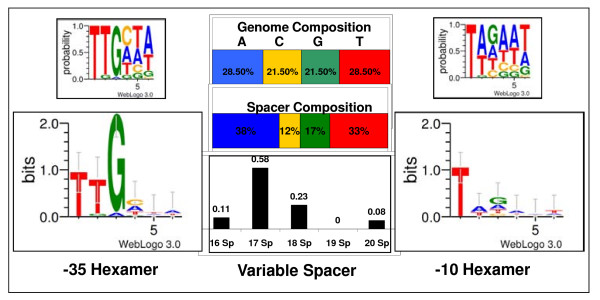
**Visualization of the M2 duration HMM**. The top WebLogos illustrate nucleotide frequencies in each of the hexamer positions. The bottom WebLogos convert the frequencies to bits of information.

The M2 duration HMM describes and quantifies the RNAP-σ^66^/DNA binding observed in the training set. A visualization of the M2 parameters is shown in Figure 3. The -35 hexamer is dominated by the initial TTG motif, while the initial T with frequent As and Ts describe the -10 hexamer. The C and G compositions (12% and 17%, respectively) of the spacer region are much smaller than those of the genome (21.5% each). Spacer lengths of 17 predominate, while spacers of length 19 are absent.

The input data file for **durahmmer **(ts1.txt) and the resulting output data file (ts1_hmm.txt) are provided as Additional Files 1 and 2. The output data file is an HMMer 2.3.2 model file which supplies the parameters of the M2 duration HMM to **hmmsearch**. Complete documentation for the contents of the file can be found in the HMMER User's Guide at . Briefly, the first 17 lines are header information with the main model section following. There are 3 lines for each of the 32 possible nodes. The first and last 6 nodes refer to the -35 and -10 hexamers, while nodes 7 through 26 refer to possible spacer positions. The first line for each node displays the contribution to the final score (multiplied by 10^3^) for the corresponding nucleotide matching A, C, G or T. The third line is particularly relevant to nodes 22 through 25, which correspond to spacer nucleotides 17 through 20. As nucleotides in these positions may or may not be present in the sequence being scored due to variable spacer length, the third line provides the odds of transitioning to another spacer nucleotide or to the -10 hexamer.

The M2 prediction equation generated by SBLR is:



Being the strongest predictor, HMM_SCORE is selected in the first step of the SBLR procedure. The prediction equation for step one is



Using a classification cutoff of P = 0.5 and setting u = 0 yields HMM_SCORE = 0.339 as the threshold for step 1 classification. At step 1, 14/26 promoters and 2220/2226 non-promoters were classified correctly. Thus, the remaining eight model variables moved 12 promoters with HMM_SCORE < .339 to promoter classification and 6 non-promoters with HMM_SCORE ≥ .339 to non-promoter classification (without altering the classification of the previous 2234 observations).

The predictor variables and their coefficients describe the verified promoters and their upstream regions. Promoters have high HMM_SCORE and low POSITION. The near upstream region is curved and unstable, whereas the further upstream region is uncurved and unstable under superhelical stress. For late-cycle genes where expression onset occurs at 24 h PI, the effect of superhelical stress is less than at other times (a positive SIDD coefficient indicates there is little destabilization of DNA under superhelical stress). The upstream characteristics may reflect transcription factor binding and/or additional interaction with the RNAP holoenzyme.

The interpretation of the positive coefficient for STABLE1_37 is more subtle. In the second step of the SBLR, four observations change from FP to TN and 5 observations change from FN to TP. The means of STABLE, STABLE1_37 and STABLE33_37 are all larger in the second group than in the first. Although STABLE33_37 shows the greatest mean difference, the most statistically significant is STABLE1_37.

### Model Exercise: Predicting Promoters for the *CT *Genome

Finally, the M2 model was used to predict promoters for the entire *CT *genome. Additional File 4 reports 479 predicted promoters in 361 unique genes, along with their HMM scores and genome locations. Thus, for 534 of the total 895 *CT *genes, this model does not find any 32-mers with a probability > 0.5. This suggests a conservative prediction that emphasizes specificity over sensitivity. Other explanatory factors may include alternate binding patterns for σ^66^, alternative σ-factors, and operon configurations.

There was a substantial overlap among predictions by different methods. Additional File 5 lists the 209 promoters (176 unique genes) co-predicted by M2 and NNPP2.2, while Additional File 6 lists the 175 promoters (162 unique genes) co-predicted by M2 and TSS-PREDICT. Additional File 7 reports the 98 promoters (90 unique genes) co-predicted by M2, NNPP2.2 and TSS-PREDICT. All predictions are for 40 = POSITION = 325, consistent with the range of the modeling procedure.

Of the 42 promoters predicted by Footy, 11 were members of the M2 training set, 4 (CT265_111, CT342_102, CT547_065 and CT606_149) were co-predicted by M2 and NNPP2.2, and 6 (CT267_097, CT269_82, CT446_245, CT546_050, CT646_071, and CT837_088) were predicted by all four algorithms.

Characteristics of the M2 genome-wide prediction can be summarized by looking at all 479 predictions, or by looking at the 361 unique genes, and selecting the predictions closest to the TLS. The two views produce similar results. Approximately 64% of predicted promoters are completely contained in non-coding upstream regions, 50% are on the positive strand, and time of activation distributes as follows: 5% hour 1, 23% hour 3, 51% hour 8, 20% hour 16 and 2% hour 24. The strand and hour distributions for all 895 genes in the genome are equivalent to the predicted promoter distributions, indicating that there is no strand or temporal preference for the predicted *CT *σ^66 ^promoters.

**Figure 4:**
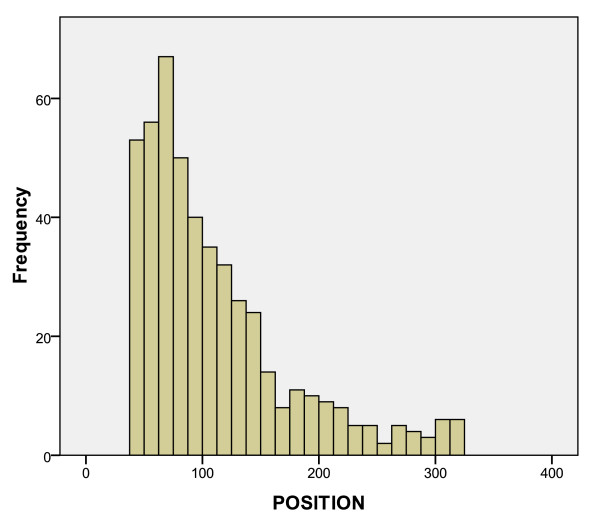
**Histogram of predicted promoter position, n = 479**. POSITION marks the 5' end of the data-file 32-mer, and is consequently ~40 nt upstream from the TSS. This distribution peaks with the 5' end around 68 nts upstream from the TLS and the TSS around 28 nts upstream from the TSS.

Figure 4 displays a histogram of predicted promoter positions. POSITION marks the 5' end of the data file 32-mer, and is consequently ~40 nt upstream from the TSS. Thus, the POSITION distribution peaks with the 5' end around 68 nts upstream from the TLS and the TSS around 28 nts upstream from the TSS. The peak and shape of this distribution closely resemble the *E. coli *histogram from Burden *et al *(2005) [11].

## Discussion

The final model produced by the iterative strategy was generated by a training set with three of the original members, CT665, CT681a and CT681b, removed. An explanation of how these three sequences differ from the remainder would be informative. The last column of Table 1 reports that CT665 and CT681 are both expressed at 8 h PI, classifying them as mid-cycle genes. Niehus *et al *(2008) [36] recently demonstrated that chlamydial promoters show a differential response to changes in DNA supercoiling that correlates with the lifecycle expression pattern. Specifically, two mid-cycle genes (8 h PI) responded to supercoiling, while three late-cycle genes (≥ 16 h PI) did not. Their experimental set included *ompA*/CT681 in the mid-cycle group and *omcA*/CT444, *hctA*/CT743 &*ltuB*/CT080 in the late-cycle group. Thus, it is likely that there exists a set of mid-cycle promoters that differ topologically from other promoters to enhance their ability to respond to supercoiling, and this may explain the anomolous characteristics of these promoters that we observed.

A possible explanation for the large number of genes without promoter predictions by the M2 model is heterogeneity requiring different models, for example for response to supercoiling. While investigating the initial model M0, we explored stepwise nominal regression, which allows for the discovery of more than two dependent variable categories. However, we did not find that a third category was substantiated. Nonetheless, we suspect that future promoter identifications may confirm the existence of more than two promoter populations for σ^66 ^in Chlamydiales.

A chief limitation of our study includes the challenge of collecting a reliable training set that was discussed earlier. We also feel that it would be advisable in future studies to relax the range of possible spacer lengths in the duration HMM for increased generalization, which might have allowed the discovery of more promoters in the whole genome analysis. Additionally, it is quite possible that there are structural features downstream from the TLS, as well as upstream, which would aid in promoter discovery. Future modeling efforts should extend the region of interest to 100 nt downstream from the TLS.

The high priority assigned to the duration HMM scores by the SBLR procedure reinforces that the duration hidden Markov model is an encouraging approach for modelling core promoters, that deserves further development. Also by implementing our model in HMMer our duration HMM is reusable, generalizable, easily adapted to other organisms and open-source. This approach explicitly incorporates spacing preferences of elements in a likelihood framework. Two natural further developments of this approach would include further iteration of the model development in *Chlamydia *using an expanded training set, exploiting computational criteria and measurements to define expanded training sets. Another possible extension would be to model extended promoter elements using further elaborations of the hidden Markov modeling framework.

The *CT *genome-wide promoter predictions and co-predictions with other algorithms provide the basis for future research in promoter identification. The fact that 20% of M2 predicted promoters were co-predicted by NNPP2.2 and TSS-PREDICT supports the validity of all three predictions. The expected confirmation of these promoters will augment the list of verified promoters. However, confirming or rejecting the predictions made by only M2 will provide more valuable information. Confirmation will strengthen the current model in a direction that diverges from *E. coli*, while rejection will add new non-promoter observations that differ from the current training set.

## Conclusion

Models M1 and M2 support the conjecture that measures of DNA biophysical criteria along with measures of RNAP σ-factor/DNA binding collaboratively contribute to a sequence's ability to promote transcription. Whereas a measure of RNAP σ-factor/DNA binding ensures a sensitive prediction, adding measures of position relative to the TLS, stability, curvature, SIDD and twist provide specificity. The stratified K-fold cross-validation of M2 indicates that the model performs well by absolute criteria as well as compared to other predictive algorithms. Additionally, there is considerable overlap between the genome-wide predictions of M2 and NNPP2.2, TSS-PREDICT and Footy.

The modeling procedure we describe here seems especially appropriate for bacterial species where the set of known promoters is limited and the genome is relatively small.

## Outlook

The model derived by the method described here is a first pass model that serves as proof of concept. The *CT *genome-wide promoter predictions, along with co-predictions by NNPP2.2, TSS-PREDICT and Footy, will allow researchers to select optimal candidates for validation mapping of transcript 5' ends by primer extension. As more chlamydial promoters are identified, the model will be updated, and a refined list of promoter predictions may be developed. More interactions among predictor variables may also be explored. A final model will provide insight into the process of chlamydial transcription initiation. Then, too, it will be possible to determine if chlamydial promoters differ significantly from those of other bacteria.

## Authors' contributions

RM participated in conceiving the study, designed the strategies, retrieved data from various websites, conducted the data analysis, performed calculations and wrote the R scripts. DO participated in conceiving the study and provided bacteriological expertise. DA participated in conceiving the study and provided modeling expertise. Research was performed under the advice and supervision of DO and DA. All authors contributed to the draft of the paper, and all authors read and approved the final manuscript.

## Supplementary Material

Additional file 1 Input data file for durahmmer used to build M2 duration HMM. Click here for file

Additional file 2 Parameters of the M2 duration HMM that were provided for hmmsearch by durahmmer, an HMMer 2.3.2 model file. Click here for file

Additional file 3 Data file used to build M2. Click here for file

Additional file 4 CT promoters predicted by M2. Click here for file

Additional file 5 CT promoters predicted by M2 and NNPP2.2. Click here for file

Additional file 6 CT promoters predicted by M2 and TSS-PREDICT. Click here for file

Additional file 7 CT promoters predicted by M2, NNPP2.2 and TSS-PREDICT. Click here for file
